# Gamma Knife Radiosurgery for Arteriovenous Malformations Using a Four-Dimensional Dynamic Volume Computed Tomography Angiography Planning System as an Alternative to Traditional Catheter Angiogram

**DOI:** 10.7759/cureus.2788

**Published:** 2018-06-11

**Authors:** Christopher P Cifarelli, John A Vargo, Todd Tenenholz, Joshua D Hack, Grenaville Guthrie, Jeffrey S Carpenter

**Affiliations:** 1 Neurological Surgery, West Virginia University School of Medicine/Ruby Memorial Hospital, Morgantown, USA; 2 Department of Radiation Oncology, West Virginia University School of Medicine, Morgantown, USA; 3 Department of Radiation Oncology, West Virginia University, Ruby Memorial Hospital, Morgantown, USA; 4 Radiology, West Virginia University School of Medicine/Ruby Memorial Hospital, Morgantown, USA; 5 Radiology, West Virginia University School of Medicine/Ruby Memorial Hospital, Morgantown , USA

**Keywords:** arteriovenous malformation, gamma knife, radiosurgery, four-dimensional cta

## Abstract

Background

Gamma knife radiosurgery (GKRS) remains a critical intervention in the long-term management of arteriovenous malformations (AVMs). For planning a treatment, identification of the nidus is essential, and it is dependent on high-resolution blood flow imaging, usually in the form of a traditional angiogram. The development of dynamic 320-slice computed tomography (CT) angiography has offered a noninvasive alternative to intra-arterial fluoroscopic imaging, and it is capable of providing equivalent temporal resolution. In this study, we describe the feasibility of using four-dimensional CT angiography (4D-CTA) in GKRS planning for AVM treatment and a comparative analysis with a traditional angiogram.

Methods

A retrospective review was performed on AVM patients treated via GKRS with a 4D-CTA prior to the day of treatment, on the day of treatment, or with a day-of-treatment angiogram. Treatment times, along with total times in the Leksell® coordinate frame G, were obtained from the medical records. The frame-on time was calculated by subtracting the treatment time from the total time starting from application to removal, and the statistical analysis was performed across groups using analysis of variance (ANOVA). All treatments were performed on the Perfexion™ model with a dynamic flow imaging procured via a 320-slice CT scanner or traditional angiography platform.

Results

Some 27 patients underwent a total of 29 GKRS procedures for AVM treatment at our institution between September 2011 and January 2017. Mean age at the time of treatment was 35.5 (6-65) years, and male:female ratio was 5:4. Some 12 patients had 4D-CTA performed prior to the day of treatment, eight patients had the same CTA completed after frame placement on the day of treatment, while seven patients underwent traditional angiography. The mean frame-on times of each group were 190, 336, and 426 minutes, respectively (p < 0.0001). No procedures were aborted based on the image quality.

Conclusions

4D-CTA is an effective tool in identifying the AVM nidus for GKRS planning. These studies can be performed prior to the day of treatment, allowing for a significant reduction in frame-on time and eliminating the risk of angiogram complication on the day of GKRS.

## Introduction

The appropriate treatment of cerebral arteriovenous malformations (AVMs) has been the subject of considerable debate over the past decade [1]. Risk stratification analysis of unruptured versus ruptured AVMs has prompted an in-depth discussion regarding not only the best treatment modality options, but also the primary determination to offer any form of treatment [1-2]. Despite these controversies, gamma knife radiosurgery (GKRS) continues to serve a critical function in the primary treatment of the newly diagnosed AVMs as well as the residual disease following surgical intervention [3-4]. 

Paramount to the success of GKRS treatment of AVMs is precise identification and obliteration of the nidus. The classical approach to such a treatment requires a two-dimensional (2D) cerebral angiogram after application of the Leksell® coordinate frame G (Elekta AB, Stockholm, Sweden), followed by a contrast-enhanced three-dimensional (3D) magnetic resonance image (MRI). In a patient population that has already completed a diagnostic digital subtraction angiography (DSA), this treatment-associated study is not only redundant from an imaging perspective, but also exposes the patients to the risks of additional contrast agent, catheter port site injury, as well as intracranial hemorrhage, albeit at historically low rates [5-6]. Modern imaging advances, including dynamic CT angiography on 320- and 640-slice four-dimensional (4D) scanners, offer a novel mechanism for capture of blood flow imaging with temporal resolution rivaling fluoroscopic capture and offer a safe and effective diagnostic alternative to traditional angiography [7]. Originally employed in cardiac imaging, the use of four-dimensional CT angiography (4D-CTA) has evolved to encompass diagnosis of cerebral AVMs and dural arteriovenous (AV) fistulas [8-9]. In addition, to avoid any potential DSA-associated complications, these 4D-CTA image sets provide the AVM nidal target in a 3D space for direct transfer into the planning software. 

In the present study, we review a single-institutional experience using 4D-dynamic volume CTA in GKRS of AVMs as an alternative to catheter-based angiography for the day of service (DOS) flow study used in treatment planning and identify a novel workflow for reducing the time required for the patients to remain immobilized in the Leksell® coordinate frame G.

## Materials and methods

Study design and patient population

**Figure 1:**
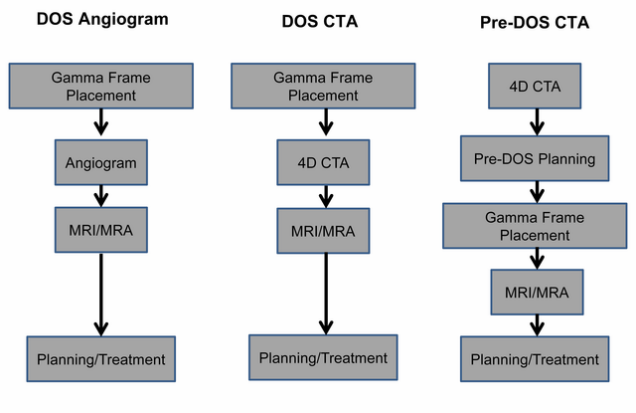
Radiosurgery AVM treatment workflow. Patients were treated by one of three mechanisms, with either an angiogram or 4D-CTA performed after frame placement on the day of service (DOS angiogram, DOS CTA) or with a CTA performed within four weeks prior to the day of treatment (pre-DOS CTA).

An IRB-approved retrospective review of the patients who received GKRS treatment for cerebral AVMs from 2011 to 2017 was performed. All the patients had a confirmatory diagnostic flow study (DSA, CTA) performed prior to GKRS. Spetzler-Martin grades were assigned to each AVM by a neuroradiologist. Three separate workflows were used for GKRS imaging and treatment (Figure 1). 

Two of the treatment paradigms included all DOS imaging (DSA or CTA) while the third distinct workflow included a pre-DOS CTA performed within the preceding 30 days of treatment. 

AVM treatment imaging 

**Figure 2:**
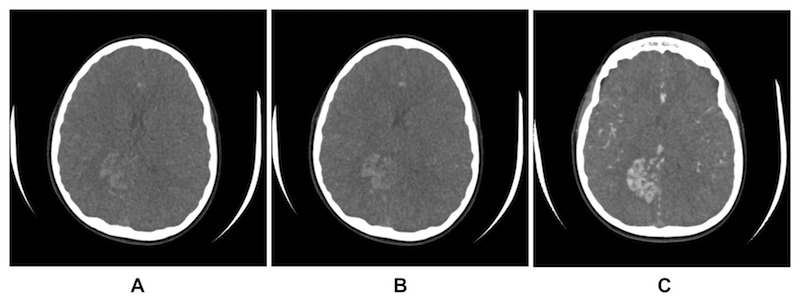
Time-resolved CTA (4D-CTA) for nidus identification. A representative case of treatment planning in an 11-year-old patient using pre-DOS 4D-CTA to identify the earliest filling phase (A) through last peak arterial phase and maximal intensity projection (MIP) images (B and C, respectively).

Dynamic flow imaging was performed for treatment targeting using either the Aquilion ONE (Toshiba Corporation, Tokyo, Japan) 320-slice CT scanner or the Artis Angiography (Siemens Healthcare, Malvern, PA) platform. The patients undergoing a 4D-CTA on the Aquilion ONE scanner were imaged using 80-120 kV, 100-240 mAs, 320 slice × 0.5 mm collimation with acquisition of three to four frames per second. Whole brain volumes were acquired and reviewed by the neuroradiologist and the treatment team (neurosurgeon/radiation oncologist) for identification of the earliest filling phase of the AVM nidus (Figure 2). 

For patients undergoing cerebral angiogram on the DOS, right common femoral artery access was secured using a 5 Fr catheter via a standard Seldinger technique. The AVM visualization was achieved following manual injection of a contrast agent and biplanar image acquisition (two to five frames per second) on the Siemens Artis angiography system.

GKRS technique

**Figure 3:**
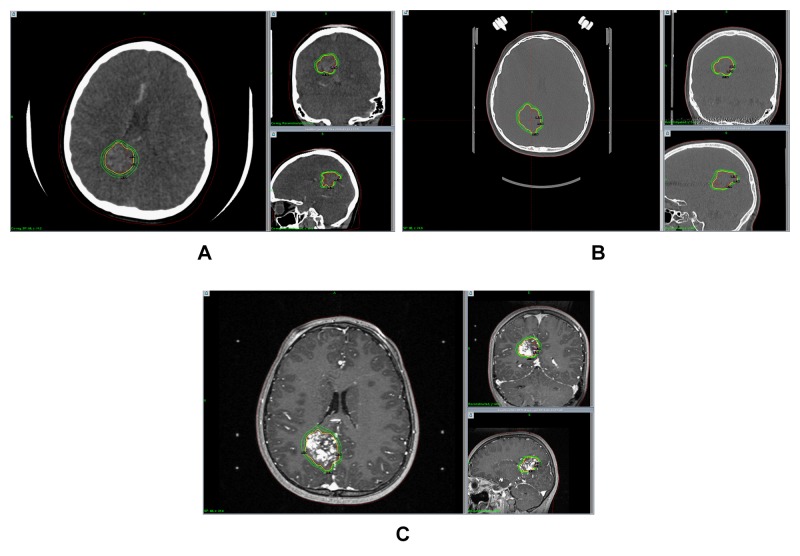
Sequential target delineation and contouring for GKRS treatment. The DICOM source volume images from the earliest filling phase of the 4D-CTA are used for contour development in GammaPlan® (A). On the day of service, the 4D-CTA, and the associated target contour, are co-registered to the volumetric CT head (B). The postgadolinium T1 and time of flight (TOF) MRI sequences are used for contour verification and further refinement as needed (C).

 Leksell® coordinate frame G placement was performed with either moderate sedation or general anesthetic. All patients were imaged with a DOS MRI/MRA and either a diagnostic cerebral angiogram or a 4D-CTA. The digital imaging and communications in medicine (DICOM) formatted images were uploaded to the GammaPlan® software (Elekta AB, Stockholm, Sweden) where a conformal target representing the nidus was developed using the dynamic flow study (Figure 3A). A volumetric noncontrast CT head was performed for skull definition and used for image fusion with CTA when 4D-CTA was acquired (Figure 3B). Target contouring and refinement with a gadolinium-enhanced MRI was used as a confirmation of nidus location (Figure 3C). All treatments were completed on the Perfexion™ Gamma Knife system (Elekta AB, Stockholm, Sweden) utilizing 4, 8, and 16 mm collimators. Frames were removed immediately at the completion of the treatment, regardless of the level of anesthesia.

Calculation of “in frame” time

The precise time of frame application and removal were noted in each medical record. The total treatment beam-on time was subtracted from the total frame application time to determine the “in frame” time for each patient, independent of the treatment length and expressed in minutes. All cases for the treatment of AVM were treated as the first case of the day, eliminating any excess "in frame" time that could occur when multiple patients were treated with GKRS within the same day. 

Determination of obliteration 

The follow-up imaging was performed at regular intervals (three to six months) with a combination of CTA, MRA, or a catheter angiogram. The time to obliteration (TTO) was determined by calculating from the DOS of GKRS to the date of the first imaging study demonstrating absence of fistulous flow. Formal angiograms were used as the gold-standard confirmatory examination. In cases where the preceding CTA or MRA appeared to demonstrate obliteration and this was subsequently by a catheter angiogram, the date of the preceding test was used for calculation.

Statistical analysis

Data are expressed as mean and standard error of the mean for the continuous variables of age, target volume, maximal dose, margin dose, and “in frame” time. Statistical significance was determined via one-way analysis of variance (ANOVA) with multiple comparisons with post-hoc analysis (Fisher’s LSD) using GraphPad Prisim V6.07 (GraphPad Software Inc, LaJolla, CA). Statistical significance was determined at the level of p < 0.05.

## Results

Patient demographics and radiosurgical treatment parameters

Some 27 patients underwent a total of 29 GKRS procedures for AVM treatment at our institution between September 2011 and January 2017. Mean age at the time of treatment was 35.5 (6-65) years, and the overall male:female ratio was 5:4. Spetzler-Martin grades ranged from I to V. Analysis performed on the subsets of patients according to the three treatment workflow groups is summarized in Table 1. No statistically significant differences were noted amongst these groups (p > 0.05). The maximal dose received across all the patient workflow categories ranged from 32.1 to 47 Gy, while the mean margin doses ranged from 16 to 23 Gy with all prescriptions at the 50% isodose line, both without any significant differences across the workflow categories (Table 1).

**Table 1:** Patient demographics and GKRS treatment data. No significant differences were seen in the patient or AVM characteristics across each different workflow group based on ANOVA. The only significant difference (p < 0.0001) was seen in the calculated “in-frame” time, consisting of the total frame time minus the beam-on treatment time.

	Pre-DOS CTA	DOS CTA	DOS Angiogram	p
Patient demographics	*N* = 12	*N *= 8	*N* = 7	
Mean age (years)	32.4 (11-55)	43.1 (17-63)	27.4 (6-65)	0.16
Gender (M:F)	2:1	1:1	3:4	0.591
Target volume (cm^3^)	4.65 ± 1.39	7.25 ± 1.89	6.54 ± 1.90	0.867
Spetzler-Martin grade	II-7; III-3; IV-2	I-1; II-3; III-2; IV-2	I-1; II-1; III-3; IV-1; V-1	0.176
Treatment parameters				
Maximal dose (Gy)	37.2 (32.1-40.5)	39.2 (36.1-44.9)	38.1 (32.2-47.0)	0.503
Margin dose (Gy)	18.5 (16-20)	19.3 (18-22)	18.9 (16-23)	0.674
Frame time (minutes)	190 ± 13.5	336 ± 31.3	426 ± 37.8	<0.0001

**Reduction of “in frame” time

The AVM patients treated with a GKRS using a pre-DOS CTA for the identification of the nidal target had a significant reduction of “in frame” time (190 ± 13.5 minutes) compared to a DOS angiogram (426 ± 37.8 minutes; p < 0.0001) and DOS CTA (336 ± 31.3 minutes; p = 0.0016). Furthermore, a comparison of DOS CTA and DOS angiogram did demonstrate a more modest nonsignificant reduction of mean “in frame” time by 89.3 min (p = 0.0816) (Table 2).

**Table 2:** Statistical analysis of frame times. Using the traditional DOS angiogram approach as the reference, the mean difference in adjusted frame time was found to be significantly less (p < 0.0001) in the group of patients with pre-DOS imaging and pre-planning.

	Frame time (min)	Mean difference (DOS Angio Ref.)	p	95% CI
Pre-DOS CTA	190 ± 13.5	235	<0.0001	146-324
DOS CTA	336 ± 31.3	89.3	0.0816	-9.95-189
DOS Angiogram	426 ± 37.8	Ref.	Ref.	

General anesthetic time

A total of five out of the total 27 patients reviewed received general anesthesia for frame placement and subsequent GKRS treatment, including 57% of pediatric patients. Of those patients receiving general anesthesia, the patients in the pre-DOS CTA group had a mean “in frame” time and general anesthetic time, of 193 ± 29 minutes while the DOS angiogram group had an anesthetic exposure time of 261 ± 69 minutes, a difference that did not reach statistical significance (p = 0.471).

Time to obliteration of AVM

Of the 27 patients treated with GKRS for AVM, follow-up imaging data was available for 23 patients, with 11 patients demonstrating complete obliteration. Among those with radiographic evidence of obliteration, the time from treatment to obliteration was not significantly different among the treatment groups, with an overall mean of 885 days (2.4 years) (Table 3).

**Table 3:** Time to obliteration. Of the 23 patients with continued follow-up, a total of 11 had radiographic evidence of obliteration [angiogram (five), CTA (five), or MRA (one)], without significant difference in the time to obliteration.

	Mean time to obliteration (days ± SEM)	*n*	p
Pre-DOS CTA	890 ± 123	5	0.985
DOS CTA	904 ± 261	3	
DOS Angiogram	861 ± 86	3	

## Discussion

The successful use of GKRS within a wide scope of intracranial diagnoses is directly proportional to the precision with which the treatment dose is delivered and the accuracy of the pre-procedural images in identification of the target. In this regard, both Perfexion™ and ICON™ Gamma Knife systems (Elekta AB, Stockholm, Sweden) boast a QA precision check of less than 0.4 mm, while the latter is variable across imaging modalities. The need for volumetric data in AVM target delineation has traditionally required CT and MRI acquisition with correlation of the flow data from angiography [10-11]. Unfortunately, the 2D data from the DSA is of limited use in developing the 3D plan, even with precise co-registration via the Leksell® coordinate frame G [12]. Prior studies have attempted to determine the capacity of MR angiography as a potential replacement for the nidal data provided by DSA. While several studies found that medium-sized AVMs with compact niduses were adequately demarcated with MRI/MRA only, others found greater utility in MRI as an adjunctive imaging modality determining that DSA remained necessary for accurate targeting based on variances in plans to known target locations [13-14].

The development of the 4D-CTA using 640-slice acquisition allows for image capture that approaches that of biplanar fluoroscopic images, reaching 0.3-3 frames/second in comparison to 3-10 frames/second in DSA [15-16]. Coupled with the ability to rapidly image the entire volume of the head over 16 cm, the 4D-CTA remains a powerful diagnostic tool for a variety of intracranial pathologies [17-18]. Our work expands that role by assessing the feasibility of performing a high-resolution dynamic CT angiogram as an alternative to the traditional catheter-based angiography, specifically in the context of treatment planning of GKRS for AVMs.

Vascular imaging with temporally resolved 3D CT angiography is not a novel technique, having been established as an effective method for cardiovascular imaging, including coronary artery evaluation [19-20]. With regard to diagnostic cerebrovascular imaging, 4D-CTA has been shown to be a reliable means of identification of AVMs, dural AV fistula, as well as aneurysms [18, 21-22]. Here, we present the first series of AVM patients treated with GKRS using 4D dynamic volume CTA angiography for nidus localization and treatment targeting, either before or after the placement of the Leksell® Coordinate Frame G on the day of GKRS treatment. Previously, our institution has reported a single case in which the 4D-CTA was used as a DOS study for treatment planning, including the use of titanium frame pins, creating a significant image artifact [23]. This artifact has been well documented by other groups utilizing only CT-based treatment planning as an alternative to MRI [24-25]. As there is no frame present in the pre-DOS CTA images, any artifact potentially obscuring nidus identification has been subsequently avoided.

With the development of the ICON system, gamma knife radiosurgery has experienced its first trend toward frameless treatment strategies, as already embraced by Linac-based platforms [26-27]. As more patients receive treatment via this system, we are likely to see further reduction in the need for frame-based radiosurgery, especially in large lesions already treated with dose- or volume-fractionated SRS [28-29]. In our study, treatment remains a frame-based procedure, but the use of the 4D-CTA as part of the planning process in advance of frame placement demonstrates a significant reduction of “in frame” time. Although not quantifiable, we were also provided increased time for image analyses with CTAs performed prior to the day of service, insuring that all members of the treatment team, including the neuroradiologists, had the opportunity to review the images and develop a consensus opinion on the optimal target site. 

In addition to the reduction in nontreatment frame time, we examined the effect of the pre-DOS CTA planning on the amount of general anesthetic provided during treatment. Of the 27 patients treated, only five received general anesthesia, including nearly 60% of the pediatric AVM patients reviewed in this study. Across the treatment groups, pre-DOS CTA and DOS-angiogram, we did find a reduction in mean general anesthetic time (193 ± 29 minutes versus 261 ± 69 minutes). Yet, these data were not significant, likely based on the limited total number of patients (n = 5). Given the link between procedure duration and risks of anesthetic complications in pediatric patients undergoing general anesthesia for radiotherapy cases, this reduction in total anesthesia time may importantly reduce such risks [30]. 

**Figure 4:**
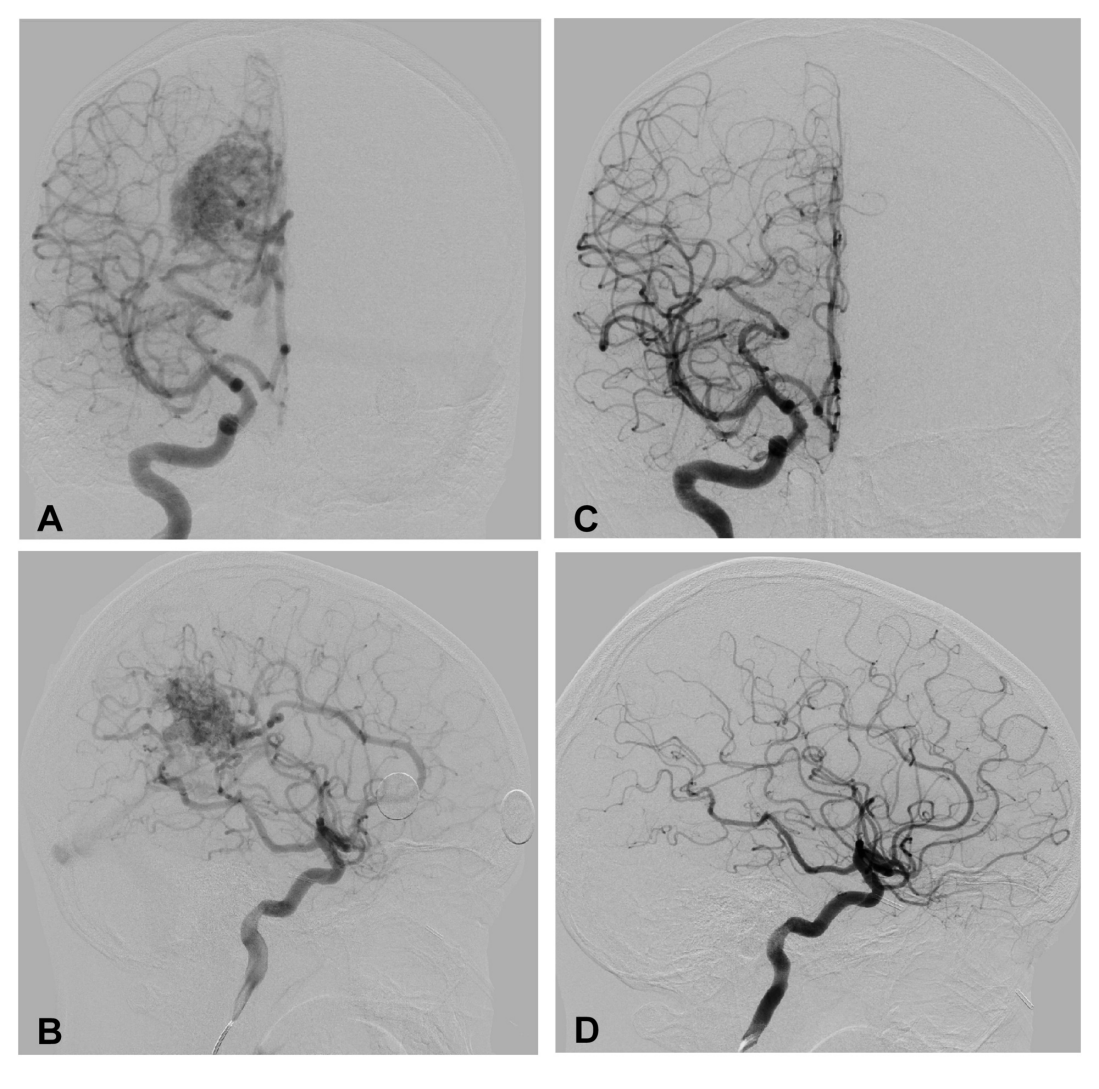
Example of AVM obliteration using 4D-CTA pre-planning technique. A diagnostic cerebral angiogram was used for initial diagnosis and characterization of the AVM architecture (A and B). Following GKRS, serial MRI/MRA examinations were performed every six months until obliteration was suspected. At 18 months following GKRS, repeat angiogram demonstrated complete obliteration of the Spetzler-Martin Grade III AVM (C and D).

The limitations of this study begin with its single institution, retrospective nature. The limiting factor in expanding this work on a multi-institutional basis is obviously imaging equipment availability, recognizing that 320- and 640-slice CT scanners are not in widespread use. Perhaps the greatest limitation of this work is the fact that has been designed as a feasibility study only, not aimed at comparing obliteration rates, although the TTO did not vary across the different workflows (Table 3). In Figure 4, we present a representative radiographic timeline to obliteration over 18 months following GKRS in an 11-year-old patient who underwent the pre-DOS CTA treatment.

Once again, angiography remained the gold standard diagnostic instrument for initial diagnosis and confirmation of obliteration once MRI/MRA and/or CTA indicated its use. Given the natural history of AVMs, including those treated with GKRS, we anticipate having the ability to report the complete obliteration follow-up data in the pre-DOS CTA and DOS CTA patients as the three-year post-treatment time frame passes. 

This study does not support the use of 4D-CTA as a replacement imaging modality in lieu of traditional cerebral angiography for lesion diagnosis. We recognize that the accurate characterization of the complex architecture of the majority of AVMs often requires the use of traditional catheter angiography, especially in higher grade Spetzler-Martin lesions. Moreover, the test of cure in our patient population remains catheter-based angiography. Yet, despite the continued importance of DSA in AVM management, we assert that the data support the utility of 4D-CTA in GKRS planning for AVM and would benefit from further study. Finally, the use of a DOS volumetric CT scan for image merging with the pre-DOS CTA may be applicable to AVMs treated via additional SRS treatment platforms, such as CyberKnife. Unlike the DOS angiogram that utilizes gamma frame data assimilation, the skull contours used in our work presumably would be applicable to frameless devices, although our preference for optimal treatment outcomes remains GKRS at our institution.

## Conclusions

4D-CTA offers a reliable means of nidus delineation for GKRS planning in AVM treatment. Performing 4D-CTA prior to the day of treatment offers a significant reduction in frame time. Preliminary results demonstrate similar times to obliteration among CTA-assisted planning cases and those using traditional catheter-based angiograms.
